# Effect of Interleukin-15 Receptor Alpha Ablation on the Metabolic Responses to Moderate Exercise Simulated by *in vivo* Isometric Muscle Contractions

**DOI:** 10.3389/fphys.2019.01439

**Published:** 2019-11-26

**Authors:** Emanuele Loro, Cholsoon Jang, William J. Quinn, Joseph A. Baur, Zoltan P. Arany, Tejvir S. Khurana

**Affiliations:** ^1^Department of Physiology, Pennsylvania Muscle Institute, Perelman School of Medicine, University of Pennsylvania, Philadelphia, PA, United States; ^2^Department of Chemistry, Lewis-Sigler Institute for Integrative Genomics, Princeton University, Princeton, NJ, United States; ^3^Institute for Diabetes, Obesity, and Metabolism, Perelman School of Medicine, University of Pennsylvania, Philadelphia, PA, United States; ^4^Department of Physiology, Institute for Diabetes, Obesity, and Metabolism, Perelman School of Medicine, University of Pennsylvania, Philadelphia, PA, United States; ^5^Department of Medicine, Penn Cardiovascular Institute, Perelman School of Medicine, University of Pennsylvania, Philadelphia, PA, United States

**Keywords:** muscle, exercise, interleukin-15 receptor alpha, metabolomics, *in vivo* stimulation, exercise mimetics, metabolism, fatty acid oxidation

## Abstract

Lack of interleukin 15 receptor alpha (IL15RA) increases spontaneous activity, exercise capacity and protects from diet-induced obesity by enhancing muscle energy metabolism, suggesting a role as exercise mimetic for IL15RA antagonists. Using controlled *in vivo* muscle stimulation mimicking moderate exercise in normal and Il15ra^–/–^ mice, we mapped and contrasted the metabolic pathways activated upon stimulation or deletion of IL15RA. Stimulation caused the differential regulation of 123 out of the 321 detected metabolites (FDR ≤ 0.05 and fold change ≥ ±1.5). The main energy pathways activated were fatty acid oxidation, nucleotide metabolism, and anaplerotic reactions. Notably, resting Il15ra^–/–^ muscles were primed in a semi-exercised state, characterized by higher pool sizes of fatty acids oxidized to support muscle activity. These studies identify the role of IL15RA in the system-wide metabolic response to exercise and should enable translational studies to harness the potential of IL15RA blockade as a novel exercise mimetic strategy.

## Introduction

Regular exercise is a safe and effective disease modifier for managing obesity and many preventable chronic diseases related to energy metabolism, as it promotes fat utilization, weight control, and ameliorates insulin sensitivity (Borghouts and Keizer, 2000). In practice, however, the people who would benefit the most from exercising often have exercise intolerance or limited mobility. In the United States, only 52% of adults perform some kind of aerobic activity (Source: Behavioral Risk Factor Surveillance System BRFSS, Centers for Disease Control and Prevention, Atlanta). From a translational perspective, making the beneficial effects of exercise universally available in the form of pharmacological intervention (exercise mimetics) is an important goal. Research on exercise mimetics has led to the identification of genes and pathways that, when modulated, can increase exercise performance and simulate the exercised condition (North et al., 1999; Lagouge et al., 2006; Quinn et al., 2013; Fan and Evans, 2017; Amoasii et al., 2019). Activators of PGC1a, AMPK and PPARδ are well-known examples of exercise mimetics (Narkar et al., 2008; Matsakas and Narkar, 2010) that can promote oxidative metabolism. Precise control over the experimental conditions is necessary because energy metabolism is influenced by multiple factors such as circadian rhythms (Dyar et al., 2018), intensity (Horowitz and Klein, 2000; van Loon et al., 2001), and duration of exercise (Romijn et al., 1993). While studies in human subjects have the highest translational value, they often face issues with recruitment, compliance and controlling for lifestyle factors, disease features or co-morbidities (Anziska and Inan, 2014). Animal research, on the other hand, offers controlled and homogeneous experimental conditions and the possibility to adopt appropriate genetically engineered models, *in vivo* physiological techniques and high-throughput analyses.

Increasing evidence in humans and mice suggests that another strategy to mimic exercise is by modulating the cytokine interleukin 15 (IL15) or its primary binding partner, IL15 receptor alpha (IL15RA). IL15 and IL15RA have a well-recognized immunological role in mediating the activation of T and natural killer cells, and constitutive ablation of either of them leads to blunted immune response to pathogens (Lodolce et al., 1998; Schluns et al., 2005). However, consistent with their widespread tissue distribution, IL15/IL15RA have also been shown to participate in the regulation of energy processes of a variety of tissues (He et al., 2010), including brain (Wu et al., 2010; Nguyen et al., 2017), bone (Djaafar et al., 2010; Loro et al., 2017), adipose tissue (Sun and Liu, 2015; Lacraz et al., 2016), and muscle (Pistilli and Quinn, 2013; Loro et al., 2015, 2018; O’Connell et al., 2015). We and others have shown that ablation of IL15RA in mice increases spontaneous activity, exercise capacity and protects against diet-induced obesity (He et al., 2010; Pistilli et al., 2011; Loro et al., 2015). Similar to the muscles of endurance-trained athletes (van Loon and Goodpaster, 2006), fast muscles from mice lacking IL15RA (Il15ra^–/–^) have higher intramuscular triglycerides (IMTG) content compared to controls. We have proposed that the ability to use IMTG stores efficiently enhances Il15ra^–/–^ recovery capacity after exercise. Supporting our hypothesis, we found that lipases, fatty acid binding proteins and the AMPK/ACC pathway were significantly activated in Il15ra^–/–^ fast muscles (Loro et al., 2015, 2018). Little is known, however, about the impact of these changes on the metabolome of resting and exercised muscles.

In the current study, we have developed an experimental model of moderate-intensity isometric exercise using *in vivo* stimulation of the sciatic nerve. Following stimulation, we have performed a detailed metabolomic analysis of the changes evoked by contraction in extensor digitorum longus (EDL) muscles. Finally, we have compared the obtained contraction-induced metabolomic signature with the changes occurring in the muscles of Il15ra^–/–^ mice, highlighting the metabolic reactions associated with their exercise performance.

## Results

### Establishment of the *in vivo* Moderate Muscle Stimulation Protocol

**FIGURE 1:**
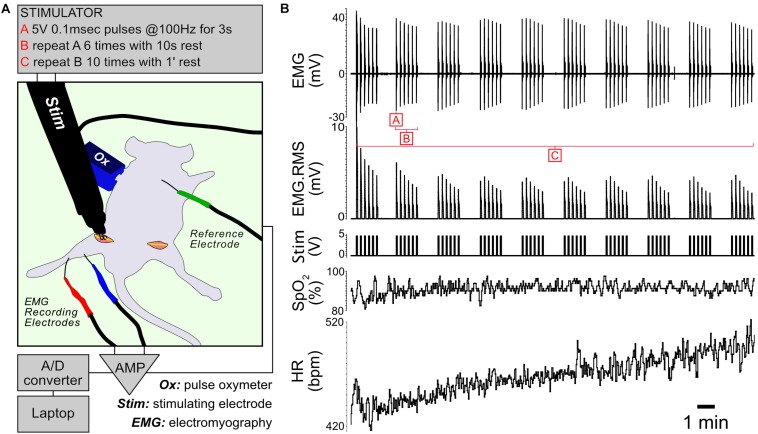
*In vivo* stimulation protocol. **(A)** Diagram of the experimental setup. The unstimulated limb underwent a sham procedure before dissecting the muscles of interest. **(B)** Representative traces showing EMG, RMS (root mean square) of the EMG trace, stimulation, capillary oxygen saturation and heart rate measured during the experiment.

Muscle contractile activity is supported by a cascade of biochemical and metabolic events fueled by the conversion of energy substrates to ATP. To study the effects of controlled exercise on muscle energy metabolism, we developed an *in vivo* stimulation protocol that mimics moderate exercise in fed anesthetized mice (Figure 1). The protocol consists of controlled bursts of muscle activity alternating with periods of rest, repeating over 22 min. When applied to control and Il15ra^–/–^ mice, the stimulation evoked the simultaneous contraction of all the muscles of the anterior and posterior compartments of the targeted limb. For each animal, one limb underwent stimulation while the contralateral was used as unstimulated control. The protocol did not induce complete muscle exhaustion, as indicated by the only partial decrement of EMG amplitude and root mean square (RMS) (Figure 1B). Consistent with moderate exercise, peripheral capillary oxygen saturation (SpO_2_) remained stable, while heart rate (HR) increased moderately as a consequence of the repeated stimulation (Figure 1B).

### Generation of C57BL/6-II15ra^–/–^ Mice With Fatigue-Resistant Muscles and Higher Spontaneous Activity

**FIGURE 2:**
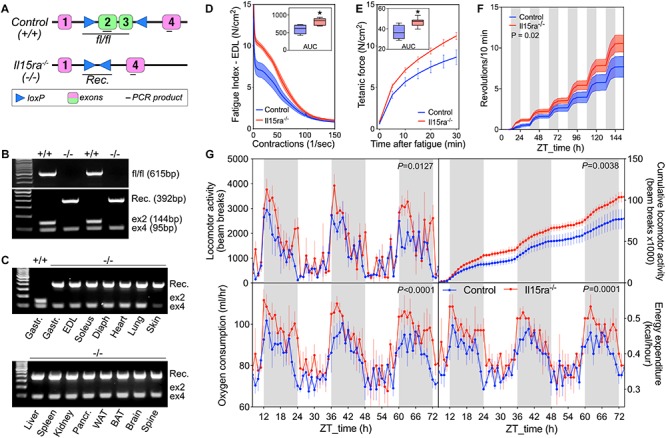
Generation of C57BL/6-Il15ra^–/–^ mice with enhanced exercise capacity. **(A)** Diagram of EIIA-cre-mediated genomic deletion of exons 2 and 3 of the *Il15ra* gene. **(B)** Genomic PCR for the detection of the intact Il15ra-flox alleles (615 bp), recombined genomic sequence after the cre-mediated flox excision (392 bp), exon 4 (95 bp), and exon 2 (144 bp, absent in fully recombined samples). **(C)** Genomic PCR to confirm the successful recombination in multiple tissues of Il15ra^–/–^ mice, with consequent excision of the exon 2 sequence. Representative *ex vivo* EDL fatigue **(D)** and recovery **(E)** traces of control (*n* = 4) and Il15ra^–/–^ (*n* = 6) mice. Insets show the average area under the curve (AUC). **(F)** Cumulative wheel running traces (*n* = 5 mice per group). Cumulative distributions were compared with the Kolmogorov–Smirnov test. **(G)** Representative indirect calorimetry assay of control (*n* = 5) and Il15ra^–/–^ (*n* = 3) mice. Data are represented as mean ± SEM. ^∗^*P* < 0.05 and ^∗∗^*P* < 0.01.

Previous studies performed on a B6;129×1-based Il15ra^–/–^ model had raised the concern of potential differences in background compared to the control strain used (B6129SF2/J). To address this concern, we generated a new C57BL/6-based Il15ra^–/–^ line by crossing IL15RA-flox mice to mice expressing Cre driven by the ubiquitous promoter EIIA (Figure 2A) and performed every experiment using control and Il15ra^–/–^ littermates. Genomic PCR detected the successful recombination and full excision of exons 2/3 (Figure 2B), the region coding for the IL15 binding site in IL15RA. We confirmed the successful excision in all tissues, including muscle, brain, liver, spleen and adipose tissue (Figure 2C). To test whether the C57BL/6-Il15ra^–/–^ line (from now on called Il15ra^–/–^) retained the previously described muscle phenotype, we compared EDL muscles from Il15ra^–/–^ and littermate controls in terms of isometric strength (Supplementary Table S1), resistance to fatigue (Figure 2D) and recovery capacity (Figure 2E). Measurements of muscle strength and weight were similar between genotypes (Supplementary Table S1). Consistently with the previous reports, Il15ra^–/–^ EDL retained higher fatigue resistance (Figure 2D) and recovery capacity (Figure 2E), suggesting differences in energy metabolism. *In vivo*, Il15ra^–/–^ retained the overall normal circadian rhythm of locomotor activities, although they were more active than controls when tested for cumulative wheel running activity (Figure 2F) and beam breaks (Figure 2G, – top panels), with higher oxygen consumption and energy expenditure especially during the dark phase (Figure 2G, – bottom panels).

### Metabolomic Analysis of EDL Muscles

**FIGURE 3:**
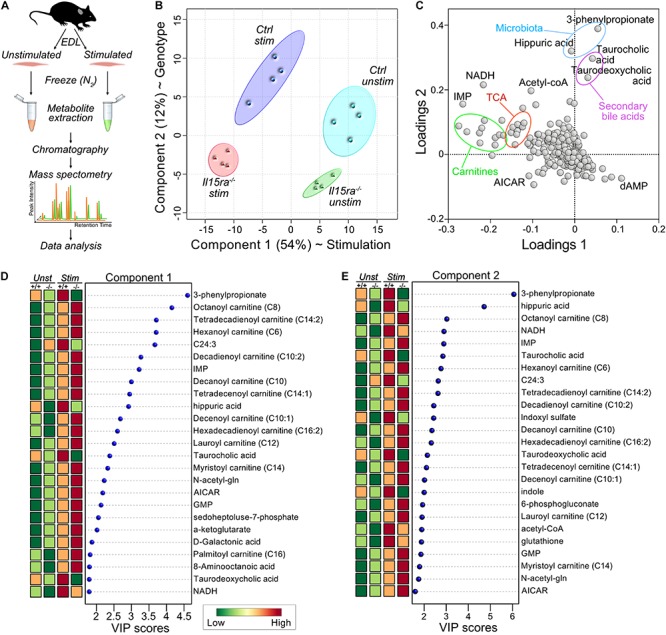
Supervised multivariate analysis. **(A)** Diagram of the experimental pipeline adopted for the study. **(B)** Partial Least Square Discriminant Analysis (PLS-DA) plot of the metabolomics data. **(C)** Loadings plot for the principal components selected by PLS-DA. **(D,E)** Ranked representation of Variable Importance in Projection (VIP) scores for the features contributing to the variance of component 1 **(D)** and 2 **(E)**, according to PLS-DA.

Because changes in transcriptomics or proteomics are unlikely to drive the acute response to a single bout of muscle stimulation by themselves, our approach focused on the analysis of muscle metabolome. We performed targeted metabolomics on unstimulated and stimulated EDL muscles (Figure 3A). A total of 321 metabolites were detected and identified by GC-TOF MS analysis (Supplementary Table S2). To model the effects of stimulation and genotype, we applied a supervised partial least squares discriminant analysis (PLS-DA). The significance of class discrimination, assessed using a permutation test based on prediction accuracy, was calculated to be *P* = 0.007. The PLS-DA model distinguished between the contribution of two main components, accounting respectively, for 54 and 12% of the total variance. Component 1 best separated the effect of stimulation, while component 2 separated the effect of genotype (Figure 3B). A loading plot representation (Figure 3C and Supplementary Table S3) highlighted the contribution of carnitine species and TCA cycle intermediates to the variance associated with component 1 (stimulation), and the contribution of gut microbiota metabolites to the variance of component 2 (genotype). A ranked representation of the 25 highest variable importance projection (VIP) scores for each component confirmed the contribution of carnitines, fatty acids, and TCA intermediates to the variation associated with component 1 (Figure 3D and Supplementary Table S4). Carnitines and fatty acids were also among the 25 highest VIPs for component 2 (Figure 3E and Supplementary Table S4), suggesting an interaction between genotype and stimulation.

### Effects of Moderate-Intensity Stimulation

**FIGURE 4:**
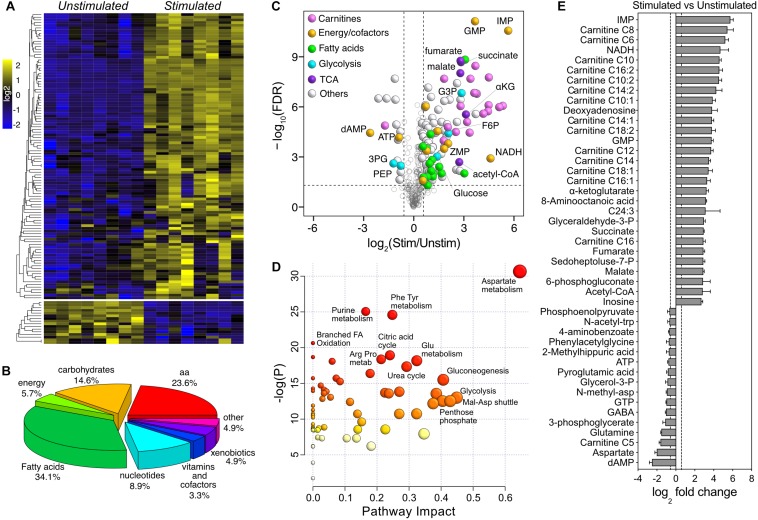
Univariate analysis of the effect of moderate exercise. **(A)** Heatmap of log_2_-transformed, row-wise normalized intensities for metabolites with FDR-adjusted *P* < 0.05 and fold change > ±1.5 (123 of 321). **(B)** Pie chart representation of the metabolite superclasses significantly affected by stimulation. **(C)** Volcano plot showing the effect of stimulation. The dotted horizontal line represents the significance threshold (ANOVA, FDR-adjusted *P* ≤ 0.05, *n* = 8), while the dotted vertical lines represent the fold change cut-off (± 1.5). Significant data points are color-coded according to the different superclasses. **(D)** Pathway impact analysis of metabolites differentially regulated upon stimulation. The node color is based on the *P*-values from pathway enrichment (*y-*axis), while the size is proportional to the pathway impact value (*x*-axis). **(E)** Ranked representation of the 45 most differentially regulated metabolites upon stimulation (*n* = 8).

By applying a two-way mixed ANOVA analysis (Genotype × Stimulation) with statistical significance at α ≤ 0.05 after FDR adjustment, 195 metabolites were significantly affected by stimulation (123 more than 1.5×), while only 7 were significantly different between genotypes (5 more than 1.5×) (Supplementary Table S2). Because of the magnitude of the changes associated with stimulation, we initially dropped the genotype factor and focused on the comparison between stimulated and unstimulated muscles. The majority of stimulation-induced changes were toward higher metabolite levels (107 up and 16 down – Figure 4A). The most represented metabolite superclasses were fatty acids (34.1%), amino acids (23.6%), carbohydrates (14.6%), and nucleotides (8.9%) (Figure 4B). Consistent with the PLS-DA model, carnitines were predominant within the group of upregulated metabolites (Figure 4C). Pathway analysis suggested that the most represented pathways impacted by stimulation were amino acids metabolism (Asp – *P* = 4.20E-12; Phe, Tyr – *P* = 1.79E-09; Glu – *P* = 1.03E-06; Arg, Pro – *P* = 8.57E-07), purine metabolism (*P* = 1.13E-09), citric acid cycle (*P* = 5.00E-07), urea cycle (*P* = 2.30E-06), glycolysis (*P* = 1.36E-04) and oxidation of branched-chain fatty acids (*P* = 1.10E-06) (Figure 4D). Due to the lack of Kegg identifiers for the fatty acids in our dataset, both measures of enrichment and impact on the entries associated with fatty acids metabolism were likely underpowered. A ranked visualization of the most significantly regulated metabolites highlighted the important role of TCA and purine metabolism in the response to stimulation in fast muscles (Figure 4E). The most differentially regulated metabolites in the dataset were inosine monophosphate (IMP, 54×, *P* = 2.34E-11) and deoxyadenosine monophosphate (dAMP, −5.7×, *P* = 3.10E-05). IMP and dAMP are intermediates in the reactions catalyzed by adenylate kinase (which converts 2 ADP – >ATP and AMP) and myoadenylate deaminase (AMP or dAMP – >NH_3_ and IMP), which are activated during medium to high-intensity exercise in response to ATP utilization (−1.8×, *P* = 5.85E-05) and changes in pH (Sahlin et al., 1989; Tullson and Terjung, 1991, 1999). Metabolism of amino acids such as glutamine, glycine, and aspartate plays an essential anaplerotic role in muscle recovery by providing intermediates for the TCA, e.g., by converting aspartate (−4×, *P* = 2.69E-07) to fumarate (7×, *P* = 1.48E-09) (Gibala et al., 1997). The moderate exercise-mimicking stimulation also decreased the creatine-phosphate/creatine ratio by ∼50% and caused a 1.6× accumulation of intramuscular lactate in both genotypes. Together, these results show that the activation of oxidative metabolism following the application of our *in vivo* stimulation protocol is consistent with a bout of moderate-intensity exercise.

### A Schematic Overview of the Effects of Stimulation on Muscle Metabolome

**FIGURE 5:**
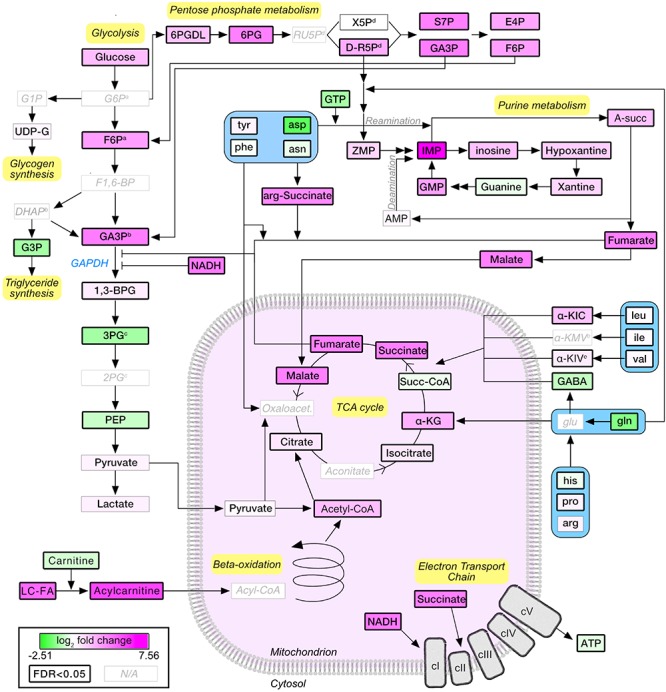
Schematic representation of the metabolic pathways regulated upon moderate exercise. Node colors are proportional to the log_2_-normalized fold change (stimulated vs. unstimulated). Statistically significant nodes (FDR-adjusted *P*-value ≤0.05) have thick borders. Metabolites that were not detected are labeled in light gray. G6P, glucose-6-phosphate; F6P, fructose-6-phosphate; F1,6-BP, fructose-1,6-bisphosphate; GA3P, glyceraldehyde-3-phosphate; 1,3-BPG, 1,3-biphosphoglycerate; 3PG, 3-phosphoglycerate; 2PG, 2-phosphoglycerate; PEP, phosphor-enol pyruvate; DHAP, dihydroxyacetone phosphate; G3P, glycerol-3-phosphate; LC-FA, long-chain fatty acids; α-KG, α-ketoglutarate; α-KIC, α-ketoisocaproate; α-KMV, α-ketomethylvalerate; α-KIV, α-ketoisovalerate; 6PGDL, 6-phosphoglucono-delta-lactone; 6PG, 6-Phosphogluconate; RU5P, ribulose-5-phosphate; X5p, xylulose-5-phosphate; S7P, sedoheptulose-7-phosphate; E4P, erythrose-4-phosphate; A-succ, adenylosuccinate; IMP, inositol-monophosphate; GMP, guanosine monophosphate. Superscript a,b,c,d,e indicate groups of metabolites which cannot be separated by the LC-MS approach used.

A pathway diagram of the main muscle energy metabolites affected by stimulation (Figure 5) suggested that, upon moderate exercise, glucose derived from increased uptake and glycogenolysis was shunted from glycolysis to the pentose phosphate pathway, generating reducing equivalents and ribose-5-phosphate that entered the purine nucleotide pathway as ZMP (also known as AICAR; 3.78×, *P* = 2.77E-04), a potent activator of AMPK and a known regulator of energy metabolism (Ezagouri et al., 2019). Pyruvate levels were not affected by stimulation, suggesting that the increased acetyl-CoA and TCA cycle intermediates were mainly derived from fatty acid degradation and anaplerotic reactions. Interestingly, glycolytic intermediates accumulated upstream of GAPDH. GAPDH enzymatic activity may be allosterically inhibited by multiple mechanisms, including nitrosylation in presence of high levels of NADH (Mohr et al., 1996) (25.95×, *P* = 1.05E-03), or by increased concentrations of fumarate (Blatnik et al., 2008) (7.48×, *P* = 1.48E-09). The application of our *in vivo* stimulation protocol allowed us to map the metabolic consequences of a single bout of moderate intensity exercise, highlighting the important contribution of fatty acid metabolism and anaplerotic reactions to muscle energetics.

### Differential Metabolic Activation in Il15ra^–/–^ Mice

**FIGURE 6:**
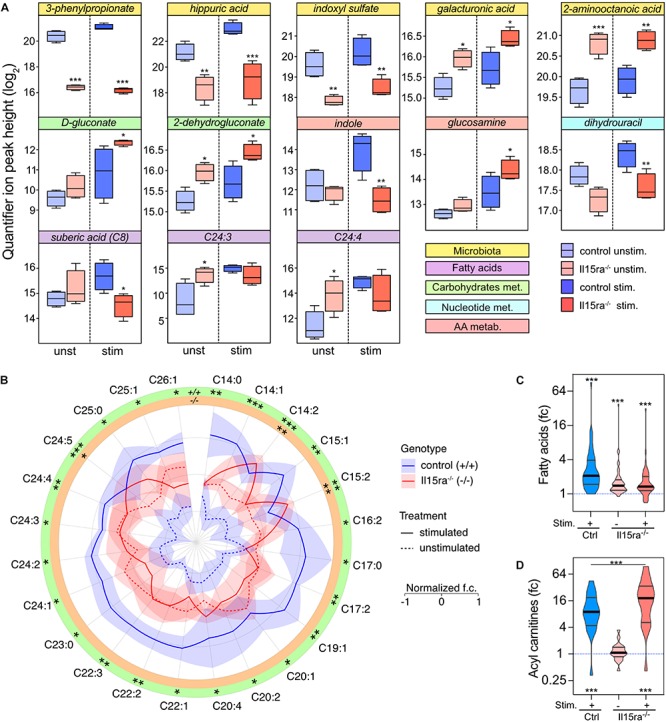
Univariate analysis of the effect of genotype. **(A)** 2-way ANOVA with multiple comparisons of metabolites with significant interaction between stimulation and genotype. Data are represented as mean ± SEM. **(B)** Radar chart representation of the levels of fatty acids significantly mobilized upon stimulation in control (blue lines) and Il15ra^–/–^ (red lines) EDL muscles. The fold changes of each fatty acid have been normalized (mean 0 and variance 1) for visualization purposes. Violin plots of mobilization of the total fatty acids **(C)** and carnitine **(D)** pools detected by the assay. In panels **(C)** and **(D)**, the dashed line represents controls unstimulated. Statistical significance was calculated vs. control unstimulated using one-way ANOVA on log_2_-transformed intensities before conversion to fold change. ^∗^*P* < 0.05, ^∗∗^*P* < 0.01, and ^∗∗∗^*P* < 0.001.

Il15ra^–/–^ mice perform significantly better than controls when tested for muscle fatigability and recovery capacity. We, therefore, focused on the differences associated with genotype in unstimulated and stimulated muscles. With this approach, the number of significantly different metabolites between control and Il15ra^–/–^ muscles were 8 before and 11 after stimulation (Figure 6). Consistent with the PLS-DA model, the metabolites showing the most significant variations between genotypes were the microbiota metabolites 3-phenylpropionate, hippuric acid, and indoxyl sulfate, suggesting an impact of IL15RA deficiency on gut immune system and microbiota. Some fatty acids, as well as intermediates of the glucuronic acid pathway (part of the pentose phosphate pathway), were significantly higher in Il15ra^–/–^ than controls. In line with previous reports showing that lack of IL15RA has a role in bone homeostasis (Djaafar et al., 2010; Loro et al., 2017), glucosamine, a metabolite important for joint health, was induced 2.2-fold by stimulation and was 1.8 times higher in the stimulated Il15ra^–/–^ than stimulated controls (Figure 6A). The AMPK activator ZMP trended to be higher in Il15ra^–/–^ than in control muscles (1.88× in unstimulated and 2.36× in stimulated muscles). Of 48 detected fatty acids, 24 were significantly higher after stimulation in control muscles (Figure 6B). In Il15ra^–/–^ unstimulated muscles, the basal pool size of long-chain fatty acids (LC-FA) was higher than in controls, but did not change after stimulation (Figures 6B,C). Given the previously described enhancement of FA uptake into Il15ra^–/–^ isolated muscle mitochondria (Loro et al., 2015), we considered whether the pool of acyl-carnitines was significantly elevated in Il15ra^–/–^ compared to control muscles. The levels of 19 detected acyl-carnitines (C2–C20) increased significantly upon stimulation and were higher in Il15ra^–/–^ compared to control muscles (Figure 6D).

**FIGURE 7:**
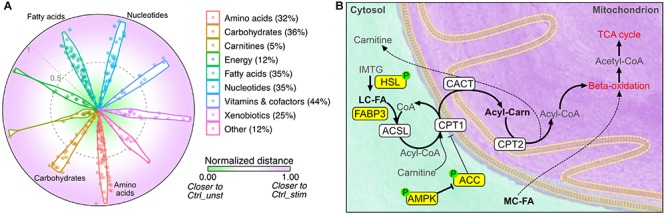
Comparison between unstimulated Il15ra^–/–^ and stimulated controls. **(A)** Radar chart representation of the Euclidean distance between unstimulated Il15ra^–/–^, unstimulated control muscle and stimulated control muscle for the metabolites in each superclass. Values were rescaled to a range from 0 (close to unstimulated control) and 1 (close to stimulated control). Percentage number indicate the ratio of metabolites in Il15ra^–/–^ muscle that were similar to stimulated control. **(B)** Diagram summarizing the effects of lack of IL15RA on muscle fatty acid metabolism. Yellow nodes indicate enzymes significantly upregulated/activated in Il15ra^–/–^ muscle, according to previous publications by our group (Loro et al., 2015, 2018). Thick arrows represent metabolic reactions that are enhanced in Il15ra^–/–^ muscle.

To obtain a visual indication of whether IL15RA ablation can function as exercise mimetic, we then calculated the Euclidean distance between the levels of each metabolite in Il15ra^–/–^ muscles and in the unstimulated and stimulated control conditions. We found that approximately 30% of the metabolites detected in Il15ra^–/–^ unstimulated muscle, including fatty acids, amino acids, and nucleotides, were closer to the levels found in stimulated control muscles (Figure 7A, distance closer to 1).

Taken together, these results demonstrate that lack of IL15RA primes basal muscle energy metabolism to a state consistent with a moderate exercise condition. In such a state, the previously described activation of lipases and the AMPK pathway (Loro et al., 2015) cooperates with the increased availability of fatty acids to promote reliance on fatty acids and support the higher energy expenditure and exercise capacity (Figure 7B).

## Discussion

In this study, we examined the metabolic responses to a single bout of moderate isometric muscle contraction in normal mice and in mice with increased spontaneous activity and fatigue-resistance induced by the ablation of IL15RA. Our primary goal was to draw a network of the metabolic reactions activated by muscle contraction, and then identify the pathways contributing to the beneficial effects of lack of IL15RA on exercise capacity and energy metabolism. We found that (1) moderate muscle contraction activates oxidative and anaplerotic reactions; (2) consistent with the proposed exercise-mimetic effects, lack of IL15RA promotes the reliance on FAO independently from muscle activity.

The application of stimulation-evoked muscle contraction has some limitations, as it only partially recapitulates the complexity of tissue-specific and systemic reactions taking place during exercise. However, it offers superior control of frequency and intensity of the evoked contraction, therefore eliminating sources of variance such as anxiety and other behavioral factors, typically present during other *in vivo* protocols (e.g., treadmill, forced-swim test). In addition, it preserves systemic circulation of metabolites from other tissues (e.g., adipose, liver, gut). While the role of IL15 in adipose tissue lipolysis to support muscle activity was previously described (Quinn et al., 2009; Pierce et al., 2015), the differential regulation of gut microbiota-derived metabolites in stimulated IL15RA muscles is a novel finding originated from our approach. It would be interesting to use additional knockouts models to address how tissue-specific IL15RA deletion alters the response to muscle activity in adipose tissue, gut microbiome and liver. It should be considered, however, that the presence of circulating IL15RA (Bergamaschi et al., 2012) can challenge the interpretation of such studies.

The effects of lack of IL15RA are more evident in fast muscles, where it promotes fatigue-resistance (Pistilli et al., 2011; Loro et al., 2015), mitochondrial biogenesis and cristae complexity (Loro et al., 2018). We, therefore, focused on the metabolome of the EDL, a fast muscle which is highly recruited during ambulatory and exercise activity (Slawinska and Kasicki, 2002), and suitable for *ex vivo* evaluation with routine muscle contractility assessment protocols. From a translational perspective, future studies would be required to evaluate how our findings apply to human muscles, in which slow fibers are generally more abundant.

### Effect of the *in vivo* Moderate Muscle Stimulation

The predominantly fast EDL muscle, composed of approximately 80% type 2B-2X glycolytic fibers, responded to stimulation with a strong elevation of intermediates of oxidative metabolism. Upon stimulation, 40% of the detected metabolites were significantly changed. 13% of them were down-regulated compared to unstimulated condition. Among the downregulated metabolites were energy substrates such as ATP, GTP and amino acids. Amino acids such as aspartate and glutamine are substrates for the anaplerotic generation of TCA intermediates (e.g., succinate). Consistent with a moderate-intensity exercise protocol, the majority (87%) of the up-regulated metabolites belonged to FAO, TCA cycle, purine nucleotide metabolism, and pentose phosphate pathways. Similarly to our study, exercise in the form of spontaneous wheel running or treadmill increase lipolysis during the resting phase (Monleon et al., 2014; Ezagouri et al., 2019; Sato et al., 2019). Despite higher stimulated glucose levels (2.83×, *P* = 8.2E-04) from uptake and/or glycogenolysis), glycolytic intermediates accumulated upstream of GAPDH, suggesting that its enzymatic activity could be reduced by the accumulation of NADH (25.95×, *P* = 1.05E-03) (Mohr et al., 1996) or fumarate (7.48×, *P* = 1.48E-09) (Blatnik et al., 2008).

The most up-regulated metabolite upon stimulation was the nucleoside IMP (inosine monophosphate, 54.1×, *P* = 2.34E-11). The deamination of AMP to IMP occurs during moderately intense muscle activity and is catalyzed by the enzyme adenosine deaminase (AMPD), the first and rate-limiting step of the purine nucleotide cycle. The conversion of AMP to IMP is advantageous for the muscle, as it maintains the ATP:AMP ratio and spares adenine nucleotides from degradation to purine bases, allowing their quick reamination during muscle recovery (Sabina et al., 1980). Supporting the role of IMP and purine metabolism during muscle activity, AMPD activity was recently found to be elevated in EDL muscles of mice lacking HDAC3, accounting for their increased fatigue resistance (Hong et al., 2017). Another source of IMP derives from the conversion of glucose-6-phosphate to ribose-5-phosphate in the pentose phosphate pathway, which is then converted to ZMP (3.78×, *P* = 2.77E-4 induction by stimulation) and enters the purine metabolism cycle. During this process, the concomitant generation of NADPH may support the exercise-induced activation of antioxidant enzymes (Frasier et al., 2013). ZMP is also a potent activator of AMPK, which in turn regulates energy metabolism and exercise capacity by promoting glucose uptake and FAO (Narkar et al., 2008). ZMP-mediated AMPK activation could therefore participate in the regulation of the network of kinase reactions recently identified by phosphoproteomic investigations of exercised muscles (Hoffman et al., 2015; Potts et al., 2017).

In summary, *in vivo* muscle stimulation allowed us to map the metabolic reaction (oxidative and anaplerotic) activated to ensure the replenishment of energy substrates and the recovery from fatigue. We then compared such map with the metabolic reactions activated in Il15ra^–/–^ muscles.

### Role of IL15RA in the Metabolic Response to Stimulation

The enhanced fatigue resistance and energy expenditure (Figure 2) of Il15ra^–/–^ mice is accompanied by a significant reprogramming of muscle metabolism, with increased basal IMTG and higher AMPK activation. Such changes are independent of the exercise state and lead to higher basal mitochondrial uptake and oxidation of fatty acids in isolated mitochondria (Loro et al., 2015).

Here, we hypothesized that if IL15RA blockade has exercise mimetic effects, it would cause a shift in basal energy metabolism toward that of stimulated control muscles. Indeed, the levels of several metabolites in the unstimulated Il15ra^–/–^ muscles were intermediate between unstimulated and stimulated controls (Figure 7A). A significant effect of genotype was found in microbiota metabolites, amino acid metabolism intermediates, and in the pool sizes of fatty acids and carnitines (Figure 6). Glycolytic intermediates were similar between Il15ra^–/–^ and controls, suggesting normal muscle glucose utilization during exercise.

The higher energy output required for sustaining Il15ra^–/–^ exercise capacity is therefore due to enhanced IMTG utilization, fatty acids mobilization, higher mitochondria content (Pistilli et al., 2011, 2013) and cristae complexity (Loro et al., 2018), similarly to what has been described in the muscles of highly trained athletes (Nielsen et al., 2017). In contrast to other experimental models (e.g., overexpression of PGC1-alpha), ablation of IL15RA exerts such effects without changing muscle composition (Loro et al., 2018). It is noteworthy that such small but significant metabolic enhancements translate into clear benefits in terms of muscle contractility, fatigability, and energy expenditure during the mostly inactive light phase (ZT4-8). The magnitude of these differences will be likely bigger during the active dark phase.

The mechanisms responsible for the increased FAO in Il15ra^–/–^ could be mediated by AMPK and CPT1B activity. AMPK activation in Il15ra^–/–^ muscle was not accompanied by substantial changes in the AMP/ATP ratio, suggesting that other factors such as ZMP (elevated by 1.88× and 2.36× in unstimulated and stimulated Il15ra^–/–^) or calcium-dependent signaling could be causing AMPK phosphorylation (Funai et al., 2013). Together with changes in metabolites, protein phosphorylation is an important mediator of the circadian control of metabolism and physiology (Robles et al., 2017) and its kinetics are consistent with the timescale of events happening during a short bout of exercise (Hoffman et al., 2015; Potts et al., 2017). Hence, this study can serve as a starting point for future investigations correlating multiple parameters such as phosphoproteomic and metabolomic responses to different stimulation intensities, different phases of the day, or multiple rodent disease models.

In summary, our data show that IL15RA ablation elevates basal metabolic rates to levels consistent with moderate exercise, in turn favoring FAO reactions as the primary fuel. Together with our previously published work on exercise capacity and resistance to diet-induced obesity (Pistilli et al., 2011; Loro et al., 2015), these findings strengthen the rationale for the future development of new pharmacological exercise mimetic strategies by blocking IL15RA.

## Materials and Methods

### Mice

All animal experiments were conducted in accordance with protocols approved by the Institutional Animal Care and Use Committee of the University of Pennsylvania. Mice were housed at 22°C under a 12:12-h light-dark cycle (7:00 AM – 7:00 PM) with food and water provided *ad libitum*. All founder mouse lines were purchased from Jackson Laboratories. C57BL/6-Il15ratm2.1Ama/J (IL15RA-flox, stock number 022365) and B6.FVB-Tg(EIIa-cre)C5379Lmgd/J (EIIA-cre, stock number 003724) were crossed to obtain a progeny with ubiquitous deletion of the floxed alleles in all tissues. The correct genotype was confirmed by PCR of floxed alleles (according to Jackson protocol for strain 022365), Cre-recombinase (according to Jackson protocol for strain 003966), *Il15ra* exon 2 (included in the region deleted by EIIa-Cre, Forward 5′-ATTGAGCATGCTGACATCCG-3′, Reverse 5′- TCCAGTGGGCAACATTTGTG-3′, product size 145 bp) and *Il15ra* exon 4 (outside the region deleted by EIIa-Cre, Forward 5′-TGCAGCAACAATGACCTTGG-3′, Reverse 5′- TATGTGGGAAACTGGCCTGTC-3′, product size 96 bp). These mice were then backcrossed to C57BL/6J (stock number 000664) mice to obtain heterozygous Il15ra^±^ on a C57BL/6J background, used for the experimental breeding. Ten-week-old male control (Control) and Il15ra^–/–^ littermates were used for the experiments. Spontaneous wheel running (Columbus Instruments) was monitored for approximately 7 days during which mice were single-housed in cages with *ad libitum* access to food and water. Energy expenditure measurements were performed on a CLAMS setup (Columbus Instruments) at the Mouse Phenotyping, Physiology and Metabolism Core of the University of Pennsylvania. In line with previous studies, all *in vivo* experiments were performed between ZT4 and ZT8.

### *In vivo* Sciatic Nerve Stimulation

For *in vivo* stimulation, mice (4 controls and 4 Il15ra^–/–^) were anesthetized with 1% isoflurane. Depth of anesthesia was confirmed by toe pinch and by monitoring HR and SpO_2_ with a pulse oximeter (PhysioSuite, Kent Scientific). The sciatic nerve was exposed at both sides, crushed distally to the muscle terminations to prevent retrograde propagation of the stimuli, and connected to a custom-made bipolar stimulating electrode driven by a Grass S48 stimulator. Two recording platinum-iridium (bare 0.13 mm, coated 0.02 mm) needle-electrodes were applied at fixed distance (5 mm), one in the gastrocnemius and the other near the Achilles tendon. The ground electrode was connected to the skin of the back of the mouse. The recording electrodes were then connected to a Warner Instruments DP-311 differential amplifier and finally to an A/D converter (ADInstruments). Traces were acquired with LabChart 8 (ADInstruments). One leg of each mouse was electrically stimulated, the other was used as sham-operated unstimulated control and dissected prior to the start of the stimulation, paying attention to minimize bleeding. The EMG signal was used to monitor the efficiency and consistency of the high-intensity stimulation protocol. The sequence of stimuli was based on the protocol originally described by Baar and Esser (1999) and was previously used to investigate phosphoproteomic changes in exercised muscle of mice (O’Neil et al., 2009; Potts et al., 2017). Throughout the experiment, vital parameters were monitored with a pulse oximeter (Figure 1A). The stimulation protocol used for the study consisted of 3-s trains of 0.1 ms pulses repeating at a frequency of 100 Hz (Figure 1B, – block A). Each 3-s train was repeated 6 times, alternating with 10 s of rest (Figure 1B – block B). This stimulation pattern was repeated 10 times with 1 min of rest between each repetition, for a total duration of 22 min (Figure 1B, – block C).

Stimulated muscles were collected immediately at the end of the protocol. EDL muscles were quickly flash frozen after dissection and processed for metabolite extraction. An independent set of two control and two Il15ra^–/–^ mice (not included in the current study) was used to validate the efficacy of the stimulation protocol and the capacity of our metabolomics approach to detect changes associated with muscle activity.

### *Ex vivo* Muscle Physiology

Muscle physiological analysis was performed on isolated EDL muscles using an Aurora Mouse 1200A System equipped with Dynamic Muscle Control v.5.415 software. EDL muscles were dissected and analyzed in constantly oxygenated Ringer’s solution (100 mM NaCl, 4.7 mM KCl, 3.4 mM CaCl_2_, 1.2 mM KH_2_PO_4_, 1.2 mM MgSO_4_, 25 mM HEPES, 5.5 mM D-glucose) at 24°C. The twitch stimulation protocol applied was a single stimulus with a duration of 0.2 ms. Muscle length was adjusted to obtain the maximal twitch response and this length was measured and recorded as optimal length (L_0_). For measuring tetanic maximal force generation, the stimulus was repeated at a frequency of 120 Hz for 500 ms. 5 min were allowed between two tetanic contractions to ensure muscle recovery. For induction of fatigue, 5 min after the last maximal tetanic contraction, muscles were stimulated every second for 8 min using 40-Hz pulses lasting 330 ms. Following the fatigue protocol, a burst of 50 maximal tetanic contractions (120 Hz for 500 ms) was applied to ensure complete fatigue across all samples. The recovery protocol started 1 s after the last burst contraction. A maximal tetanic stimulation (120 Hz for 500 ms) was given every 5 min for 30 min, and the force recovery was expressed as the percentage of the maximal isometric tetanic force. Muscle cross-sectional area (CSA) of EDL muscles was calculated dividing the muscle mass by the product of the muscle density coefficient (1.06 g/cm^3^), muscle L_0_, and the fiber length coefficient (0.45 for EDL). Specific force was determined by normalizing maximum isometric tetanic force to CSA.

### Metabolites Extraction

Metabolomics was performed at Penn DRC Metabolomics Core. To extract metabolites from tissue samples, frozen tissue samples were ground at liquid nitrogen temperature with a Cryomill (Retsch, Newtown, PA, United States). The resulting tissue powder (∼20 mg) was weighed and then extracted by adding −20°C extraction solvent (40:40:20 methanol:acetonitrile:water), vortexed, and immediately centrifuged at 16,000 × *g* for 10 min at 4°C. The volume of the extraction solution (μL) was 40× the weight of tissue (mg) to make an extract of 25 mg tissue per mL solvent. The supernatant was collected for LC-MS analysis.

### Metabolite Measurement by LC-MS

A quadrupole-orbitrap mass spectrometer (Q Exactive, Thermo Fisher Scientific, San Jose, CA, United States) operating in negative or positive ion mode was coupled to hydrophilic interaction chromatography via electrospray ionization and used to scan from m/z 70 to 1000 at 1 Hz and 75,000 resolution. LC separation was on a XBridge BEH Amide column (2.1 mm × 150 mm, 2.5 μm particle size, 130 Å pore size; Waters, Milford, MA, United States) using a gradient of solvent A (20 mM ammonium acetate, 20 mM ammonium hydroxide in 95:5 water: acetonitrile, pH 9.45) and solvent B (acetonitrile). Flow rate was 150 μL/min. The LC gradient was: 0 min, 85% B; 2 min, 85% B; 3 min, 80% B; 5 min, 80% B; 6 min, 75% B; 7 min, 75% B; 8 min, 70% B; 9 min, 70% B; 10 min, 50% B; 12 min, 50% B; 13 min, 25% B; 16 min, 25% B; 18 min, 0% B; 23 min, 0% B; 24 min, 85% B; 30 min, 85% B. Autosampler temperature was 5°C, and injection volume was 3 μL. Data were analyzed using the MAVEN software.

### Metabolomics Data Analysis

Missing or equal to zero values (11/5136) were replaced with a small value, calculated as the half of the minimum detection limit (minimum positive values in the original data). The assumption of this approach is that most missing values are caused by low abundance metabolites (i.e., below the detection limit). The values were for the following metabolites: 6-phosphogluconate (1), C24:3 (2), NADH (2), acetyl-CoA (1), deoxyadenosine (4 = 2 control unstimulated and 2 control stimulated), Tetradecadienoyl carnitine C14:2 (1).

For all the analyses, metabolomics data were log_2_-transformed. Heatmap of log_2_-transformed, row-wise normalized (mean of 0 and variance of 1) values was generated with the ComplexHeatmap R package (Gu et al., 2016) considering euclidean distance and average linkage. PLS-DA and pathway analysis were performed using the Metaboanalyst online platform (Chong et al., 2018) with standard settings. PLS-DA is a supervised method that uses multivariate regression techniques to maximize the covariance between exploratory and categorical codependent variables. The significance of class discrimination was assessed using a permutation test (2000 permutations, *P* = 0.007). The variance on each component in relation to metabolites can be graphically represented as loading plots. The PLS-DA analysis also returned a VIP score. The VIP score describes the weighted contribution of each metabolite to the variation of each component. The 25 most important features in each component, as identified by PLS-DA, were plotted. Pathway representation in Figure 5, was obtained with Cytoscape 3.7.1. Euclidean distance for each detected metabolite in Figure 7A, was calculated as the absolute value of the difference between the log_2_-transformed mean values at the different conditions. The obtained distances were then rescaled to a range from 0 (unstimulated control) and 1 (stimulated control).

### Data Analysis and Statistics

Data are presented as mean ± standard error of the mean. All statistical analyses were performed in R 3.5.1 (R Foundation for Statistical Computing, Vienna, Austria^1^) using the packages lme4 (Bates et al., 2015) and lmerTest (Kuznetsova et al., 2017). Data visualization was performed with the ggplot2 R package (Gómez-Rubio, 2017) or GraphPad Prism 8 (GraphPad Software, La Jolla, CA, United States). All statistical tests were considered significant at α ≤ 0.05, unless stated otherwise. Univariate analysis of the metabolomics data was performed by 2-way ANOVA (Genotype × Stimulation) followed by the Benjamini-Hochberg FDR adjustment. To compare the effects of stimulation in the two genotypes, pairwise multiple comparisons were performed using the R package emmeans and adjusting the raw *P*-values using the Sidak method. ^∗^*P* < 0.05, ^∗∗^*P* < 0.01, and ^∗∗∗^*P* < 0.001.

## Data Availability Statement

All datasets generated for this study are included in the article/Supplementary Material.

## Ethics Statement

The animal study was reviewed and approved by IACUC of the University of Pennsylvania.

## Author Contributions

EL contributed to the conceptualization, the formal analysis, and the writing of the original draft. EL and CJ contributed to the methodology. EL, CJ, and WQ contributed to the investigation. EL, CJ, JB, ZA, and TK contributed to the reviewing, editing of the article writing. TK contributed to the funding acquisition and supervision.

## Conflict of Interest

The authors declare that the research was conducted in the absence of any commercial or financial relationships that could be construed as a potential conflict of interest.

## Footnotes

1 http://www.r-project.org/

## Supplementary Material

TABLE S1 Contractile and morphometric measurements of EDL muscles.

TABLE S2 Metabolomics dataset.

TABLE S3 Partial least squares discriminant analysis loadings.

TABLE S4 Partial least squares discriminant analysis VIP scores.
