# Supplementary material for: Effect of Interleukin-15 Receptor Alpha Ablation on the Metabolic Responses to Moderate Exercise Simulated by in vivo Isometric Muscle Contractions
Source: Front Physiol. 2019 Nov 26;10:1439. doi: 10.3389/fphys.2019.01439 (PMC6901992; doi:10.3389/fphys.2019.01439)
Supplement: TABLE S1 — Contractile and morphometric measurements of EDL muscles. [file Table_1.DOCX]

**Table 1:** Contractile and morphometric measurements of EDL muscles.

|  | Control | Il15ra^-/-^ | *P* |
| --- | --- | --- | --- |
| *EDL contractility* | N=4 | N=6 |  |
| *Twitch force (mN)* | 88.2±9.0 | 107.4±5.3 | 0.083 |
| *Twitch force (N/cm^2^)* | 4.7±0.5 | 5.7±0.2 | 0.057 |
| *Tetanic force (mN)* | 342.4±47.4 | 391.3±14.5 | 0.274 |
| *Tetanic force (N/cm^2^)* | 18.3±2.2 | 20.7±0.5 | 0.252 |
| *Twitch - TTP (ms)* | 17.8±1.1 | 19.9±0.6 | 0.107 |
| *Twitch - RFD (mN/sec)* | 12057.5±804.7 | 14121.7±659.3 | 0.083 |
|  |  |  |  |
| *EDL morphometry* |  |  |  |
| *L_0_ (mm)* | 11.1±0.3 | 11.9±0.2 | *0.049* |
| *Mass (mg)* | 9.9±0.4 | 10.8±0.4 | 0.212 |
| *CSA (mm^2^)* | 1.9±0.1 | 1.9±0.1 | 0.798 |

Values are mean±SEM. TTP, time to peak; RFD, rate of force development; CSA, cross sectional area; L_0_, muscle optimal length; CNF, centrally nucleated fibers.
